# Transcriptome Sequencing Analysis of Peripheral Blood of Type 2 Diabetes Mellitus Patients With Thirst and Fatigue

**DOI:** 10.3389/fendo.2020.558344

**Published:** 2020-11-09

**Authors:** Bohan Lv, Xueli Bao, Ping Li, Juan Lian, Yanxiang Wu, Tian An, Jing Zhang, Xiuyan Yang, Tingye Wang, Jiajian Zhu, Yuanyuan Hu, Guangjian Jiang, Sihua Gao

**Affiliations:** ^1^Traditional Chinese Medicine School, Beijing University of Chinese Medicine, Beijing, China; ^2^Third Affiliated Hospital, Beijing University of Chinese Medicine, Beijing, China; ^3^Department of Endocrinology, Tangshan People's Hospital, Tangshan, China

**Keywords:** circRNAs, lncRNAs, miRNA, T2DM, RNA sequencing

## Abstract

**Purpose:** The purpose of this study is to explore the differences in transcriptome expression profiles between healthy subjects and type 2 diabetes mellitus patients with thirst and fatigue (D-T2DM) and, in addition, to investigate the possible role of noncoding ribonucleic acids (RNAs) in the pathogenesis of D-T2DM.

**Methods:** We constructed the expression profiles of RNAs by RNA sequencing in the peripheral blood of D-T2DM patients and healthy subjects and analyzed differentially expressed RNAs.

**Results:** Compared with healthy subjects, a total of 469 mRNAs, 776 long non-coding RNAs (lncRNAs), and 21 circular RNAs (circRNAs) were differentially expressed in D-T2DM patients. Furthermore, several genes associated with insulin resistance, inflammation, and mitochondrial dysfunction were identified within the differentially expressed mRNAs. Differentially expressed lncRNAs were primarily involved in biological processes associated with immune responses. In addition, differentially expressed circRNAs may target miRNAs associated with glucose metabolism and mitochondrial function.

**Conclusions:** Our results may bring a new perspective on differential RNA expression involved in the pathogenesis of D-T2DM and promote the development of novel treatments for this disease.

## Introduction

In today's world, with the improvement of living conditions and the change of lifestyle, more and more diabetic patients are diagnosed. The number of people with diabetes is expected to reach 700 million by 2045 (1). In traditional Chinese medicine, diabetes is divided into different types according to the clinical symptoms such as fever, fatigue, sputum, and urinary frequency (2). The “dual deficiency of Qi and Yin syndrome” is a common type of diabetes, and the main symptoms of this type are dry mouth and throat and weakness with general fatigue. Similarly, a proportion of patients with T2DM are accompanied with thirst and fatigue in modern medicine (3–7). Therefore, the diagnosis and treatment of this type of T2DM (D-T2DM) have become an important issue in clinical work.

Non-coding ribonucleic acids (ncRNAs) are transcripts that cannot be translated into proteins, but are generally considered to have a role in regulating protein expression (8). ncRNAs are divided into long non-coding RNAs (lncRNAs) and circular loops RNAs (circRNAs) according to their morphology (9). lncRNAs are transcripts longer than 200 nucleotides that have been implicated in diverse biological functions, such as transcriptional and posttranscriptional regulation and chromatin modification (10–12). CircRNAs are a type of circular ncRNA discovered recently and attracted wide attention (13). In our previous research, ncRNAs have been shown to play a key role in the pathogenesis of T2DM and other endocrine diseases (14, 15). However, the role of ncRNAs in D-T2DM development remains unclear. Therefore, comprehensive detection and analysis of ncRNAs in the development of D-T2DM are critical for the prevention and treatment of D-T2DM.

In this study, we used RNA sequencing technology to construct RNA expression profiles in peripheral blood of patients with D-T2DM. Differentially expressed mRNAs, lncRNAs, and circRNAs were detected, and their functions were predicted by bioinformatics analysis, in order to discover new targets and provide assistance for the treatment of D-T2DM.

## Materials and Methods

### Ethics Statement and Information of Subjects

This research was approved by the Ethics Committee of Beijing University of Chinese Medicine (BUCM) (2017BZHYLL0105). All subjects, enrolled from the Affiliated Hospital of BUCM, agreed to participate in the study after fully understanding the purpose and procedure of the experiment. Full inclusion and exclusion criteria are listed in Table 1. The subjects were divided into two groups: D-T2DM group (ID: QYD1, QYD2, QYD3, QYD4, QYD5, and QYD6) and the healthy subjects as the control group (ID: LZC001, LZC002, LZC003, LZC004, LZC005, and LZC006). Subsequently, the fasting venous blood of all subjects were obtained and stored at −80°C until analysis.

**Table 1:** Inclusion and exclusion criteria.

	**Inclusion criteria**	**Exclusion criteria**
Subjects with D-T2DM	Diagnosed with T2DM; Diagnosed T2DM for at least 3 months; TCM diagnosed with “dual deficiency of Qi and Yin syndrome.”	Diagnosed with type 1 diabetes, secondary diabetes, gestational diabetes, or unknown type of diabetes Patients with stage III hypertension or myocardial infarction Patients with severe primary diseases Patients with serious complications, such as infection and diabetic ketoacidosis
Healthy subjects	FPG <5.6 mmol/L; Healthy and no associated symptoms of “dual deficiency of Qi and Yin syndrome.”	Subjects with a family history of diabetes Subjects with hypertension or other cardiovascular and cerebrovascular diseases Subjects currently taking medications.

### Total RNA Extraction, Library Construction, and Illumina Sequencing

According to the previous experimental methods, we extracted the total RNA and constructed the RNA library (16). Briefly, the total RNA was extracted by TRIzol reagent (Invitrogen, Carlsbad, CA, USA) and tested for purity using the NanoPhotometer spectrophotometer (IMPLEN, CA, USA). In addition, the concentration and integrity of RNA were assessed using the Qubit 2.0 fluorometer (Life Technologies, CA, USA) and the Agilent 2100 bioanalyzer (Agilent Technologies, CA, USA), respectively. Next, Ribo-Zero rRNA Removal Kit (Epicentre, USA) was used to remove ribosomal RNA (rRNA). RNA-sequencing libraries were prepared using the rRNA-depleted RNA by NEBnext ultra RNA library prep kit following the manufacturer's instruction. After that, the quality of libraries was determined using the Agilent 2100 System (NanoDrop ND-1000) and accurately quantified by quantitative real-time polymerase chain reaction. Lastly, the libraries were pooled according to the requirements of effective concentration and data volume and then sequenced on Illumina Hiseq 2000 platform.

### Quality Control and Mapping

Raw data were processed through in-house Perl scripts. In this step, the raw data were cleaned by removing reads containing adaptors, contaminants, and low-quality reads. All clean data have been submitted to Sequence Read Archive with accession number SRP274496. Additionally, the Q20, Q30, and GC content were calculated to estimate the quality of clean reads. Next, the clean reads were aligned to the reference genome (GRCh37/hg19) using STAR (v2.5.1b).

### Quantitative Analysis of Genes and ncRNAs

The FPKM of transcripts and ncRNAs were calculated by Cuffdiff (v2.1.1) (17). FPKM refers to the number of fragments per kilobase length from a gene per million fragments mapped. It considers the effect of both sequencing depth and gene length on fragments count.

### GO and KEGG Pathway Annotation

In order to understand the biological function of differentially expressed genes, we performed enrichment analysis by topGO software. Based on the newest KEGG database, the pathway analysis was performed to determine the significant pathway of the differential genes. Fisher test was used for enrichment analysis, and those with *P* < 0.05 were significantly enriched.

### Interaction Network Analysis

Cytoscape v2.8.2 software (http://www.cytoscape.org/) was used to construct the lncRNA–mRNA and circRNA–miRNA regulatory network based on the differentially expressed gene data in the blood between D-T2DM and healthy subjects.

### Statistical Analysis

This study used GraphPad Prism 7 (GraphPad Software, CA) and SPSS software (version 20.0) for the statistical evaluations. The results are expressed as mean ± SEM. Statistical differences were determined by Student independent *t*-test, and the significance was accepted at *P* < 0.05. Each experiment was repeated for three technical replicates.

## Results

### Clinical Characteristics of the Participants

In this study, six D-T2DM patients and six healthy subjects were enrolled. All patients fulfilled the diagnostic criteria for D-T2DM. There was no significant difference in age between the D-T2DM and control groups. The characteristics of all subjects are shown in Table 2. Compared with the control group, the D-T2DM group exhibited significant increases in FPG and TG levels.

**Table 2:** Characteristics of study subjects.

**Characteristics**	**D-T2DM patients**	**Healthy control**	***P***
Number	6	6	—
Male/female	3/3	0/6	—
Age (year)	53.50 ± 9.44	43.5 ± 6.02	0.054
BMI (kg/m^2^)	26.08 ± 5.48	23.00 ± 1.38	0.211
FPG (mmol/L)	8.15 ± 1.68	5.02 ± 0.39	0.005
HbA_1c_ (%)	9.78 ± 3.40	—	—
TC (mmol/L)	5.33 ± 0.92	4.79 ± 0.46	0.228
TG (mmol/L)	2.72 ± 1.51	0.93 ± 0.32	0.018
LDL-C (mmol/L)	3.19 ± 0.67	2.85 ± 0.48	0.330
HDL-C (mmol/L)	1.21 ± 0.30	1.58 ± 0.37	0.112

### Quality Assessments and Mapping Results

To construct expression profiles of mRNAs and ncRNAs of D-T2DM and healthy control, transcriptome data sets were generated by RNA-seq. Subsequently, quality control and mapping analysis were performed for the sequencing output (Supplementary Tables 1, 2). The base percentage of Q20 was >95.29%, Q30 was >88.92%, and the average mapping rate of the 12 samples was 95.21%. These results could indicate the high quality of transcriptome sequencing data with suitable mapping.

### Differentially Expressed mRNAs, lncRNAs, and circRNAs

**Figure 1:**
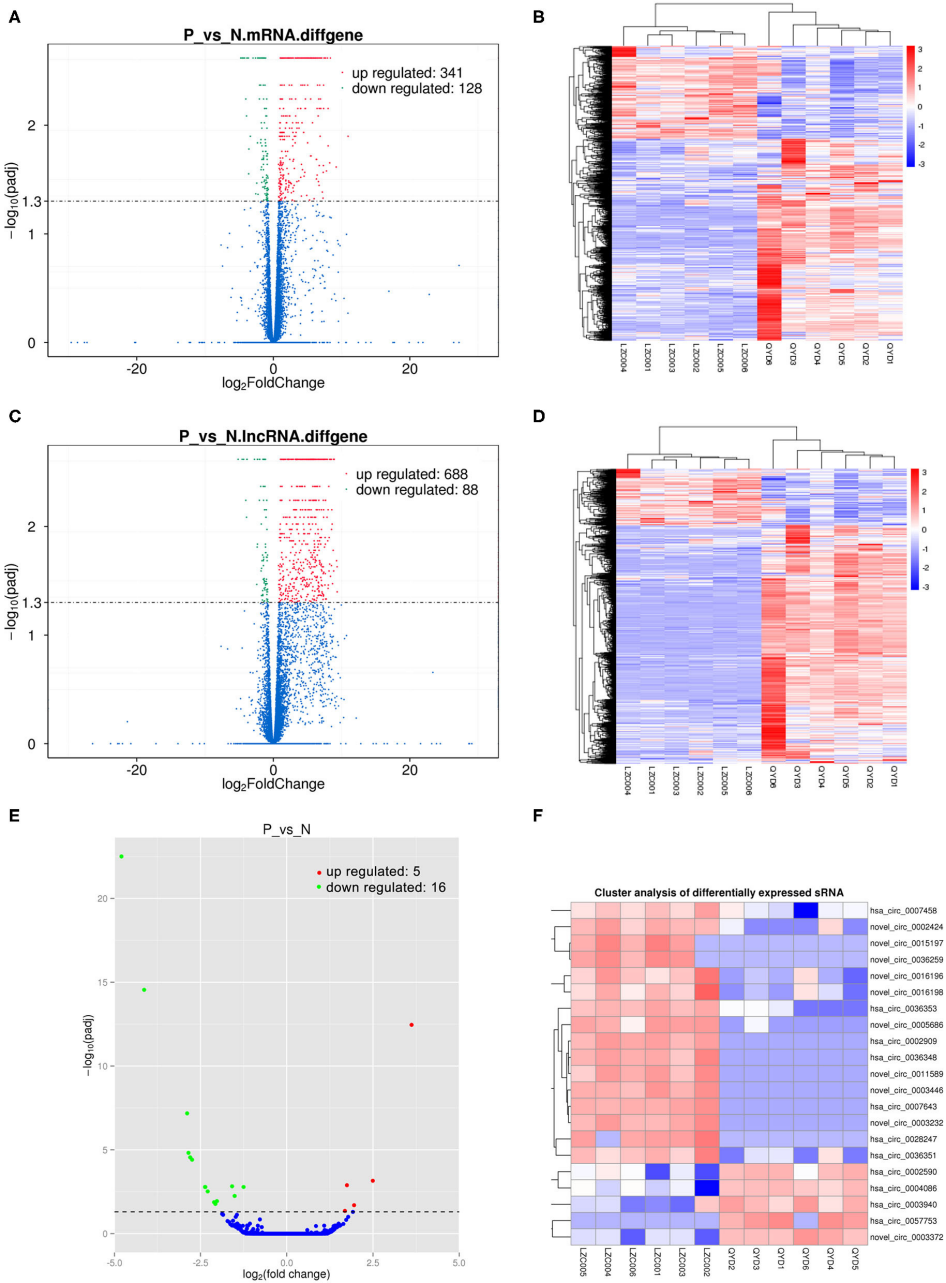
The expression profiling changes of RNAs in peripheral blood sample of D-T2DM patients. The volcano plot **(A)**, cluster map **(B)** of differentially expressed mRNAs. The volcano plot **(C)** and cluster map **(D)** of differentially expressed lncRNAs. The volcano plot **(E)** and cluster map **(F)** of differentially expressed circRNAs.

Sequencing technique was used to detect differentially expressed ncRNAs in the peripheral blood of D-T2DM patients. In Figure 1, the results showed that a total of 469 differentially expressed mRNAs (341 up- and 128 down-regulated), 776 differentially expressed lncRNAs (688 up- and 88 down-regulated), and 21 differentially expressed circRNAs (5 up- and 16 down-regulated) were detected in D-T2DM patients compared with healthy control (fold change >1.5, *P* < 0.05). Tables 3–5 list the top 10 up- and down-regulated mRNAs, lncRNAs, and circRNAs, respectively. Furthermore, Figures 1A–F shows the volcano plot, cluster map of differentially expressed mRNAs, lncRNAs, and circRNAs, respectively. Among these genes, LRRC19, GCNT3, and CKMT2 were associated with the pathogenesis of T2DM. Furthermore, mRNAs involved in regulation of mitochondrial function and lipid metabolism were significantly expressed, such as MT-ND1, MT-ND2, and OSBL6.

**Table 3:** The top 10 up-regulated and down-regulated mRNAs.

**Gene ID**	**Gene name**	**FPKM (D-** **T2DM)**	**FPKM** ** (control)**	**Log2 fold change**	***P*-value**	***P*_**adj**_**
**Up-regulated**						
ENSG00000183878	UTY	9.91899	0.0049747	10.9614	0.00045	0.0126343
ENSG00000232149	FERP1	1.22941	0.00209386	9.19759	0.00115	0.0242534
ENSG00000140297	GCNT3	1.89904	0.00381092	8.96092	0.00005	0.00240707
ENSG00000187634	SAMD11	0.752482	0.00168873	8.79958	0.00005	0.00240707
ENSG00000078795	PKD2L2	1.20053	0.00331709	8.49954	0.00005	0.00240707
ENSG00000006016	CRLF1	458.687	1.40407	8.35175	0.00005	0.00240707
ENSG00000170615	SLC26A5	1.27264	0.0041778	8.25087	0.001	0.0221781
ENSG00000136918	WDR38	29.9692	0.1154	8.02069	0.0006	0.015474
ENSG00000183562	AC131971.1	7.10184	0.0274812	8.01361	0.0002	0.00701087
ENSG00000163915	C3orf65	8.83833	0.0362467	7.92978	0.00005	0.00240707
**Down-regulated**						
ENSG00000198888	MT-ND1	74.9026	2,064.52	−4.78464	5.00E-05	0.00240707
ENSG00000074803	SLC12A1	0.23733	5.67038	−4.57848	5.00E-05	0.00240707
ENSG00000198763	MT-ND2	55.2564	1,249.02	−4.49852	5.00E-05	0.00240707
ENSG00000196557	CACNA1H	0.165957	3.35284	−4.3365	5.00E-05	0.00240707
ENSG00000212907	MT-ND4L	84.5123	1,551.15	−4.19803	5.00E-05	0.00240707
ENSG00000241404	EGFL8	0.0832157	1.35492	−4.0252	0.00015	0.00573718
ENSG00000198723	C19orf45	0.0868447	1.31358	−3.91892	0.00025	0.00822086
ENSG00000079156	OSBPL6	0.112348	1.65811	−3.8835	5.00E-05	0.00240707
ENSG00000039560	RAI14	0.0858024	1.23208	−3.84393	5.00E-05	0.00240707
ENSG00000198727	MT-CYB	73.2737	950.04	−3.69662	5.00E-05	0.00240707

**Table 4:** The top 10 up-regulated and down-regulated lncRNAs.

**Gene ID**	**Gene name**	**FPKM** ** (D-T2DM)**	**FPKM** ** (control)**	**Log2 fold change**	***P-*value**	***P*_**adj**_**
**Up-regulated**						
ENSG00000234961	RP11-124N14.3	18.5498	4.24E-287	955.523	0.00305	0.0469409
ENSG00000235790	RP11-73M7.6	1.46004	5.28E-79	260.578	0.00005	0.00240707
ENSG00000268015	CTD-2525I3.3	4.55701	0.00704305	9.33767	0.0024	0.0399329
ENSG00000268931	RP11-886P16.6	10.0556	0.0156849	9.32441	0.0011	0.0235009
ENSG00000256746	RP11-17G12.3	0.978943	0.00159989	9.25711	0.0009	0.0206451
ENSG00000232342	RP11-46O21.2	3.4293	0.00601889	9.1542	0.00005	0.00240707
ENSG00000140297	GCNT3	1.89904	0.00381092	8.96092	0.00005	0.00240707
ENSG00000260185	RP11-432I5.6	1.07359	0.0023429	8.83993	0.00125	0.0256726
ENSG00000187634	SAMD11	0.752482	0.00168873	8.79958	0.00005	0.00240707
ENSG00000254907	RP11-484D2.2	2.58357	0.00592938	8.76727	0.00065	0.0165741
**Down-regulated**						
XLOC_163941	XLOC_163941	0.250185	8.96535	−5.1633	0.00005	0.00240707
ENSG00000074803	SLC12A1	0.23733	5.67038	−4.57848	0.00005	0.00240707
ENSG00000196557	CACNA1H	0.165957	3.35284	−4.3365	0.00005	0.00240707
ENSG00000241404	EGFL8	0.0832157	1.35492	−4.0252	0.00015	0.00573718
ENSG00000198723	C19orf45	0.0868447	1.31358	−3.91892	0.00025	0.00822086
ENSG00000079156	OSBPL6	0.112348	1.65811	−3.8835	0.00005	0.00240707
ENSG00000039560	RAI14	0.0858024	1.23208	−3.84393	0.00005	0.00240707
ENSG00000224699	LAMTOR5-AS1	1.44793	9.81133	−2.76046	0.00005	0.00240707
ENSG00000115155	OTOF	0.353152	1.88766	−2.41824	0.0019	0.03413
ENSG00000236842	RP11-399K21.10	0.30283	1.55821	−2.36331	0.0006	0.015474

**Table 5:** The top 10 up-regulated and down-regulated circRNAs.

**Gene ID**	**Readcount** ** (D-T2DM)**	**Readcount** ** (control)**	**Log2 fold change**	***P*-value**	***P*_**adj**_**
**Up-regulated**					
novel_circ_0003372	146.494916	4.61683282	3.6192	1.12E-16	3.49E-13
hsa_circ_0057753	7.18354725	0	2.4945	6.01E-07	0.00070049
hsa_circ_0002590	60.9371505	7.46905994	1.9531	4.32E-05	0.020144
hsa_circ_0003940	34.4646129	8.29183529	1.7458	1.25E-06	0.0012938
hsa_circ_0004086	48.0745617	10.1103775	1.6871	9.91E-05	0.043985
**Down-regulated**					
hsa_circ_0007643	0	43.8034065	−4.793	3.37E-27	3.14E-23
hsa_circ_0002909	0	30.763914	−4.1364	6.11E-19	2.85E-15
hsa_circ_0036353	3.21483052	57.3358704	−2.8884	2.87E-11	6.69E-08
novel_circ_0011589	0	10.3931505	−2.8485	8.13E-09	1.52E-05
hsa_circ_0028247	0	13.4721127	−2.7991	1.82E-08	2.82E-05
novel_circ_0003446	0	8.46521062	−2.7445	2.91E-08	3.88E-05
novel_circ_0016196	10.5555015	34.3486988	−1.5837	1.61E-06	0.0014977
hsa_circ_0036348	0	5.86871951	−2.3608	2.30E-06	0.001646
novel_circ_0016198	71.1231467	176.561073	−1.2502	1.97E-06	0.001646
novel_circ_0036259	0	13.2664491	−2.3702	2.19E-06	0.001646

### lncRNA Target Gene Prediction

lncRNA may perform its function by regulating genes. Therefore, we predicted the biological function of lncRNA by its colocated and coexpressed genes. We set the threshold of the colocated genes to 100 kb upstream and downstream of lncRNA; mRNA gene with an absolute value of Pearson correlation coefficient >0.95 was defined as lncRNA coexpressed mRNAs (Supplementary Tables 3, 4).

### GO and KEGG Enrichment Analysis of Differentially Expressed RNAs in D-T2DM

**Figure 2:**
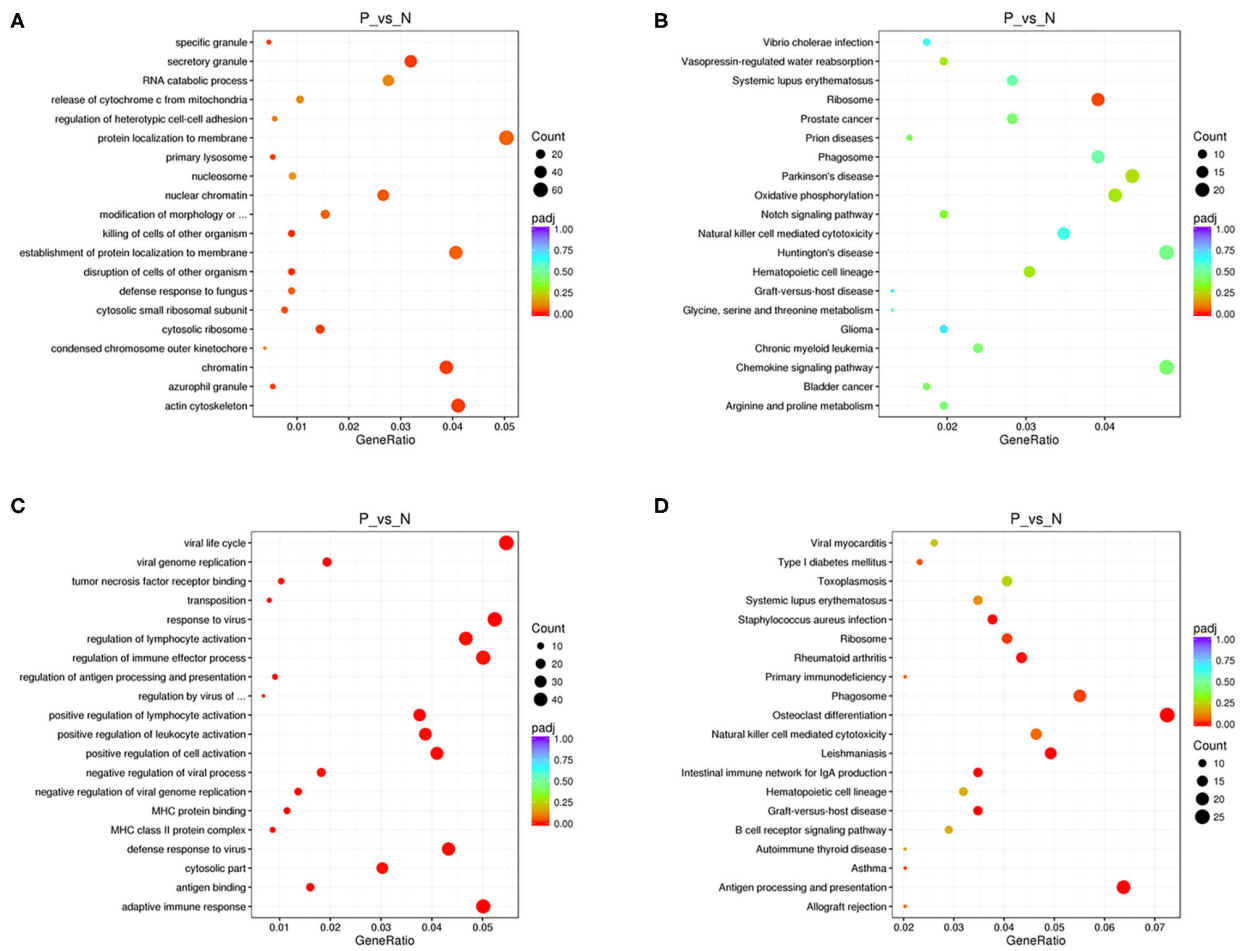
GO and KEGG analysis of mRNAs and lncRNAs in peripheral blood sample of D-T2DM patients. The scatterplots of GO enrichment results of differentially expressed mRNAs **(A)** and lncRNAs **(C)**. The scatterplots of KEGG pathway analysis of differentially expressed mRNAs **(B)** and lncRNAs **(D)**. The degree of GO and KEGG enrichment is assessed by enrichment of factors, *Q*-values, and number of genes. When the rich factor is larger, the *Q*-value is closer to zero, and the more the number of genes, the more significant the enrichment.

**Figure 3:**
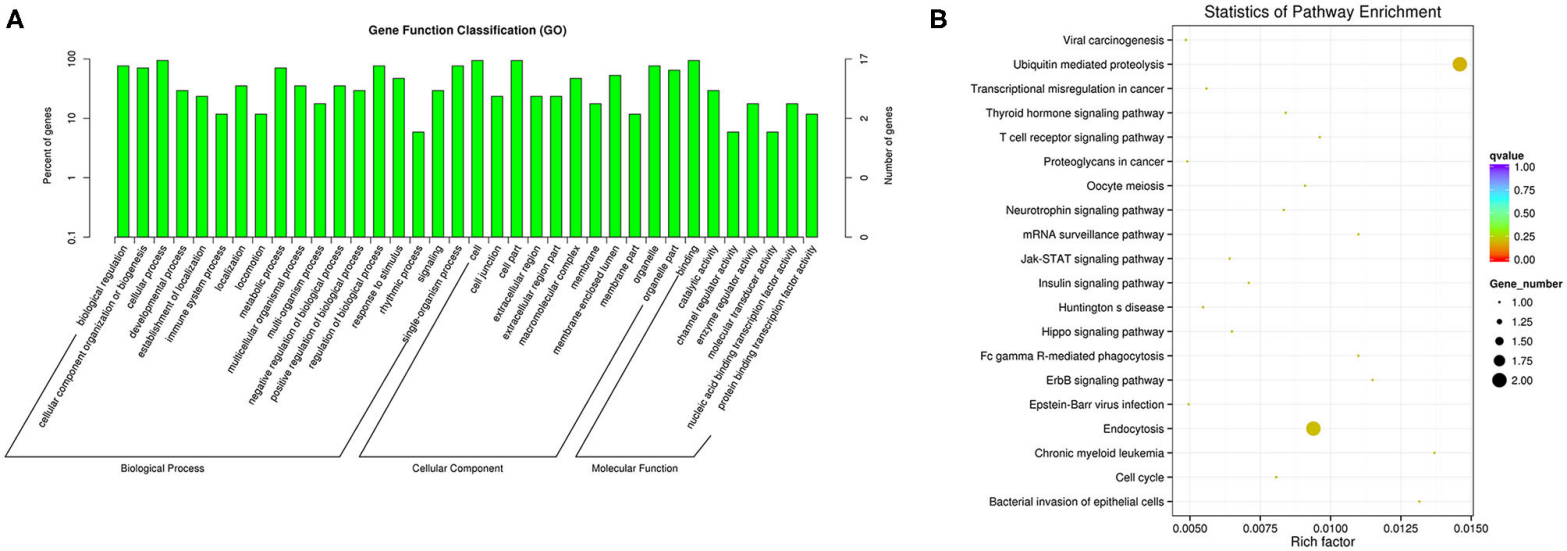
GO **(A)** and KEGG **(B)** analysis of circRNAs in peripheral blood sample of D-T2DM patients.

We performed functional enrichment analysis of differentially expressed RNAs in bioinformatics databases, including GO and KEGG analysis. As shown in Figure 2A, GO analysis of differentially expressed mRNAs revealed that the most significantly enriched biological process (BP) were disruption of cells of other organism and killing of cells of other organism, and the most significantly enriched cellular component (CC) were actin cytoskeleton, cytosolic ribosome, and secretory granule. The GO analysis of differentially expressed lncRNAs showed that the most significantly enriched BPs were adaptive immune response, positive regulation of lymphocyte activation, and response to virus, and the most noteworthy enriched CCs were MHC class II protein complex, cytosolic part, and MHC protein complex. The most significantly enriched molecular functions (MFs) were MHC protein binding, tumor necrosis factor receptor binding, and antigen binding (Figure 2C). GO analysis of differentially expressed circRNAs revealed that the most significantly enriched BPs were biological regulation, CC organization or biogenesis, and cellular process, and the most notable enrichment of CCs were cell, cell junction, and cell part. The most significantly enriched MFs were binding, catalytic activity, and channel regulator activity (Figure 3A).

KEGG analysis of differentially expressed mRNAs revealed that the most significantly enriched pathway in D-T2DM pathogenesis was ribosome (Figure 2B). The colocated mRNAs of differentially expressed lncRNAs were significantly enriched in antigen processing and presentation, osteoclast differentiation, and leishmaniasis (Figure 2D). Furthermore, differentially expressed circRNAs-derived genes were mostly involved in the ubiquitin-mediated proteolysis (Figure 3B).

### Protein–Protein Interaction Network of lncRNA Colocated mRNA Corresponding Genes

**Figure 4:**
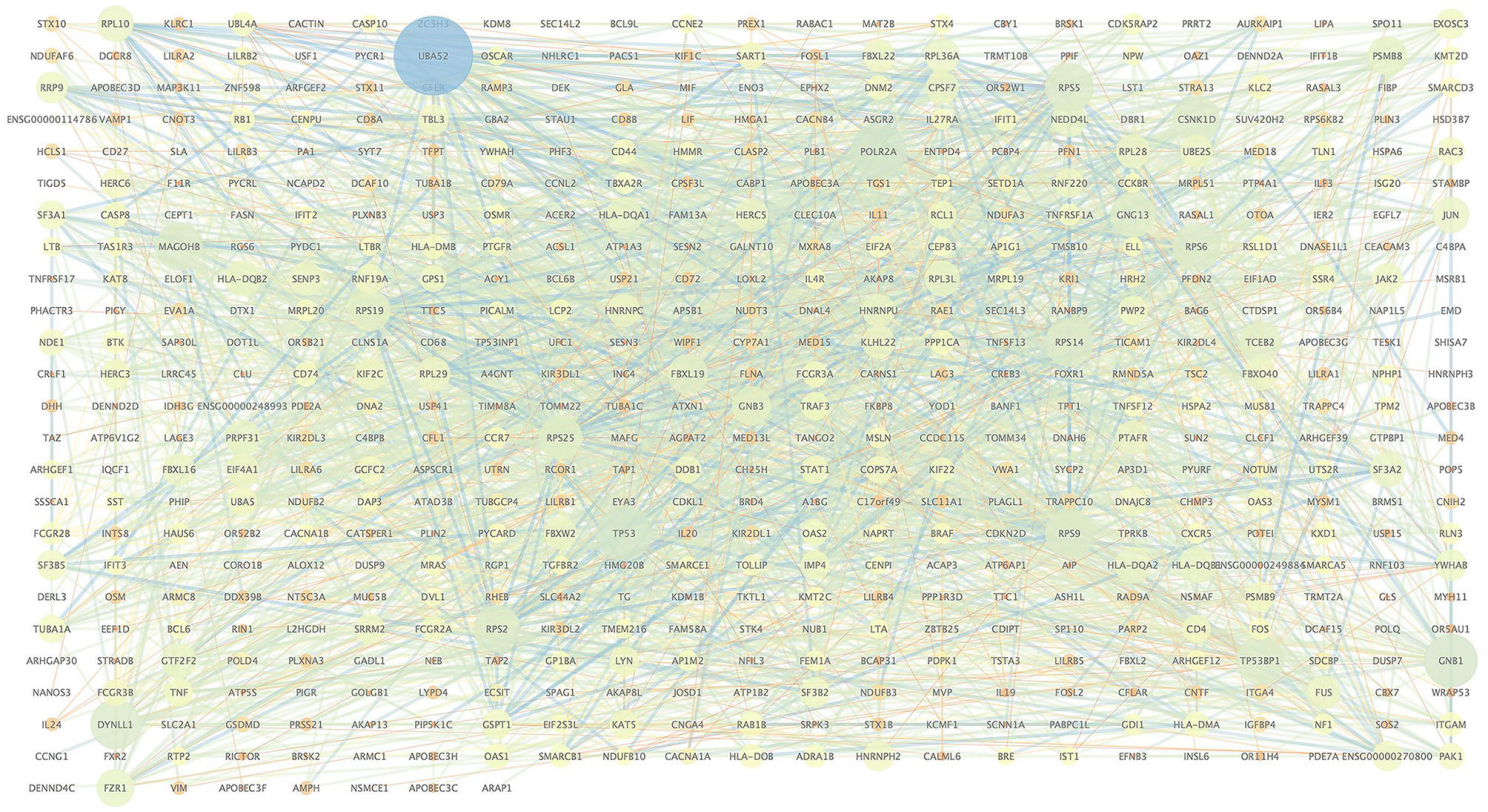
Protein interaction network of DELncRNA colocated mRNA corresponding genes.

We extracted the interaction relationship of differential gene sets to build a network forms the STRING Protein Interaction Database (http://string-db.org/) and then imported them into Cytoscape software for visual editing (Figure 4). As shown in Table 6, we listed the top 10 BP terms enriched for the genes involved in the protein–protein interaction network. UBA52 had the highest degree of network connectivity and enriched in the BP terms such as immune system process, regulation of immune response, and regulation of immune system process.

**Table 6:** Top 10 enriched biological process terms for the genes involved in the protein–protein interaction network.

**Term ID**	**Term description**	**Observed gene count**	**Background gene count**	**False discovery rate**
GO:0002376	Immune system process	193	2,370	2.91E-06
GO:0006955	Immune response	138	1,560	6.57E-06
GO:0050776	Regulation of immune response	80	873	0.0041
GO:0002682	Regulation of immune system process	113	1,391	0.005
GO:0016032	Viral process	58	571	0.005
GO:0044403	Symbiont process	63	650	0.0051
GO:0048525	Negative regulation of viral process	18	93	0.0067
GO:0051704	Multiorganism process	162	2,222	0.0076
GO:0002250	Adaptive immune response	34	280	0.0094
GO:0002577	Regulation of antigen processing and presentation	8	17	0.0094

### Regulatory Network of circRNA and miRNA

**Figure 5:**
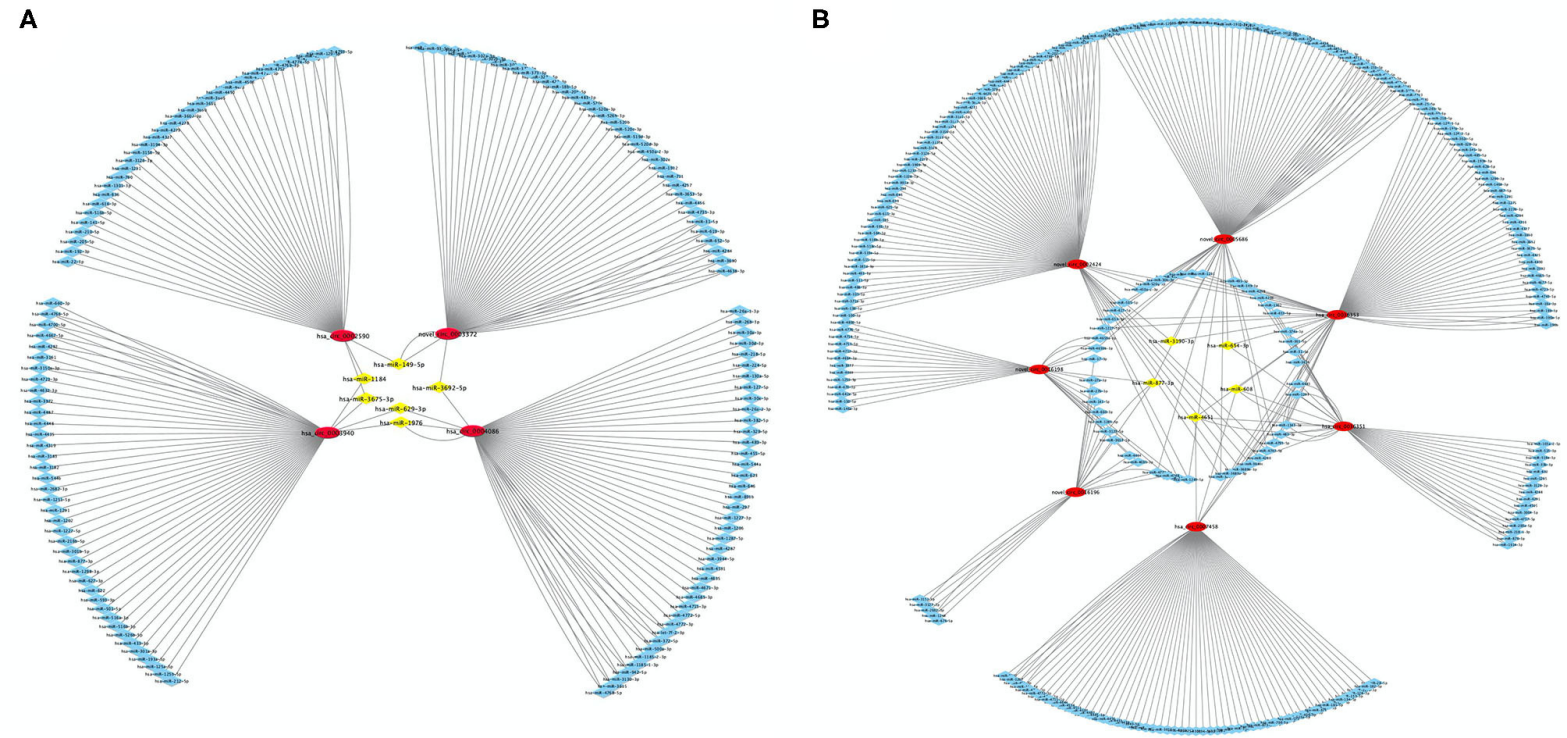
CircRNA–miRNA regulatory network analysis. **(A)** The circRNA–miRNA regulatory network analysis of up-regulated circRNA. **(B)** The circRNA–miRNA regulatory network analysis of down-regulated circRNA. Squares represent miRNAs; circles represent circRNAs.

Using differentially expressed circRNA as the center and miRNA as the target, we constructed the circRNA–miRNA regulatory network of up-regulated and down-regulated circRNAs, respectively (Figure 5). We found that 47, 38, 48, and 51 miRNA sites can be combined with up-regulated novel_circ_0003372, hsa_circ_0002590, hsa_circ_0003940, and hsa_circ_0004086, respectively; 70, 76, 80, 41, 22, 63, and 30 miRNA sites associated with down-regulated hsa_circ_0036353, novel_circ_0005686, novel_circ_0002424, hsa_circ_0036351, novel_circ_0016196, hsa_circ_0007458, and novel_circ_0016198, respectively. Among the results, MiR-877-3p was associated with T2DM and mitochondrial function and combined with novel_circ_0016196, novel_circ_0016198, and novel_circ_0005686. circRNA has_circ_0002590 and novel_circ_000372 regulated the expression of MiR-149-5p, which could regulate the insulin secretion.

## Discussion

In this study, we used sequencing technology to determine the significantly differentially expressed mRNAs, lncRNAs, and circRNAs in the peripheral blood of patients with D-T2DM. Afterward, we performed GO and KEGG pathway analysis on these differentially expressed RNAs to predict their potential biological functions. In addition, we also constructed protein–protein interaction network and circRNA–miRNA regulatory network. Our results indicated that ncRNAs may play a role in the pathogenesis of D-T2DM and provide some potential targets for the treatment of D-T2DM.

The sequencing results showed that there were 469 mRNAs, 776 lncRNAs, and 21 circRNAs significantly dysregulated in D-T2DM patients compared with healthy subjects. In up-regulated mRNAs, we have found some targets related to the pathogenesis of T2DM, such as LRRC19, GCNT3, and CKMT2. Among them, LRRC19 could activate nuclear factor κB (NF-κB) and induce expression of pro-inflammatory cytokines (18). GCNT3 was also related to inflammation (19–21). Inflammation can lead to a cluster of chronic metabolic disorders such as insulin resistance, obesity, type 2 diabetes, and cardiovascular disease (22–24). In addition, previous studies have revealed that NF-κB and its target inflammatory factor genes such as IL-1 and IL-6 played a key role in the development of insulin resistance and T2DM (25–27). CKMT2, creatine kinase, mitochondrial 2, was an effective modulator of ATP synthase–coupled respiration (28). Among down-regulated mRNAs, there were some mitochondrial genes, for instance, MT-ND1, MT-ND2, MT-ND5, and MT-ND4L. These mitochondrial genes were associated with fatty acid metabolism, mitochondrial oxidative phosphorylation, mitochondrial energy transduction, and the diabetes mellitus pathogenesis (29–31). Mitochondria are the most essential energy production organelles, supplying energy for cell metabolism in the form of ATP (32). In traditional Chinese medicine, fatigue is a major symptom of D-T2DM patients. Meanwhile, the hallmark symptom of mitochondrial dysfunction is fatigue (33). Therefore, mitochondrial dysfunction may play a role in the pathogenesis of D-T2DM. Furthermore, OSBPL6 was positively correlated with plasma levels of high-density lipoprotein cholesterol (34). SLC12A1 may play a role in glucose-induced insulin secretion (35). In general, the results indicated that the pathogenesis of D-T2DM may be related to the glucose and lipid metabolism disorders, occurrence of inflammation, and mitochondrial dysfunction.

At present, the mechanism of interaction between lncRNA and protein-coding genes is not clear. We predicted the biological function of lncRNA through its colocated and coexpressed protein-coding genes. Among the differentially expressed lncRNAs, lncRNA-SLC12A1, lncRNA-OSBPL6, and lncRNA-GCNT3 were colocated with SLC12A1, OSBPL6, and GCNT3, respectively. Therefore, these lncRNAs may play a role by regulating the genes related to glucose metabolism, lipid metabolism, and inflammation. Afterward, this study used GO and KEGG pathway analyses to analyze the biological function and pathways of lncRNAs-related genes in the peripheral blood of patients with D-T2DM. The results showed that the most enriched GO term was adaptive immune response. Previous studies have demonstrated that adaptive immune factors was recognized as important etiological components in the development of insulin resistance (36). In our future work, the specific role of lncRNAs predicted by GO and KEGG analysis in the pathogenesis D-T2DM needs to be further studied. Protein–protein interaction network represents the interaction of the protein products of the lncRNA colocated genes and was used to predict the biological function of differentially expressed lncRNAs. The results showed that the proteins were mainly enriched in BPs related to immune response. In addition, UBA52 had the highest degree of connectivity, and previous study suggested that UBA52 may be implicated in the diabetic nephropathy (37). Therefore, differentially expressed lncRNAs appear to function in the pathogenesis of D-T2DM by regulating immune response.

In order to discover the molecular mechanism of circRNAs in D-T2DM, we constructed the circRNA–miRNA regulatory network based on the sequencing data. In our results, MiR-877-3p was combined with down-regulated circRNA novel_circ_0016196, novel_circ_0016198, and novel_circ_0005686. Xie et al. (38) found that MiR-877-3p was deregulated in type 2 diabetic kidney disease. Another study revealed that MiR-877-3p exerts its effects via the Blc-2–mediated mitochondrial apoptotic pathway (39). CircRNAs generally act as an miRNA sponge to regulate gene expression. Therefore, the down-regulation of these circRNAs may improve the function of MiR-877-3p and thus mediate mitochondrial apoptosis. On the other hand, up-regulated circRNA has_circ_0002590 and novel_circ_000372 were associated with MiR-149-5p. The overexpression of MiR-149-5p could ameliorate the high glucose–induced injury in human umbilical vein endothelial cells, whereas the inhibition of MiR-149-5p could suppress cell viability, induce cell apoptosis, and inhibit insulin secretion (40, 41). In this research, the up-regulated circRNAs competitively bound with MiR-149-5p to inhibit its function. In general, circRNAs may play a role in the pathogenesis of D-T2DM by regulating the function of MiR-877-3p and MiR-149-5p, but the specific mechanisms need further research.

In this study, we have performed a comprehensive transcriptome analysis in D-T2DM patients' peripheral blood, revealing the contribution of epigenetic changes to D-T2DM. However, the present study has several limitations. First, in order to better understand the mechanism of occurrence and development of D-T2DM, it is important to compare D-T2DM with other types of T2DM, such as a milder form of T2DM. This will be the main part of our future study. Next, the major targets of insulin actions are skeletal muscle, liver, and adipose tissue rather than the blood. Therefore, our results can explain the pathogenesis of D-T2DM only partially. We anticipate the transcriptome profile of typical insulin-targeted tissues of D-T2DM patients will be investigated in the future. In addition, because peripheral blood also includes multiple cell populations and bioactive substances, further studies are required to characterize a more precise role of ncRNAs in peripheral blood of D-T2DM patients. Our results indicate that ncRNAs may exert their biological functions in the pathogenesis of D-T2DM by interacting with each other or related proteins genes. However, the mechanism of ncRNAs is complicated; thus, we will further study these predicted ncRNAs and their involved signaling pathways. Finally, it will help to reveal the internal mechanism and therapeutic targets of D-T2DM.

In conclusion, we used sequencing analysis to study the expression profile of RNAs in the peripheral blood of D-T2DM and healthy subjects. Differentially expressed mRNAs, lncRNAs, and circRNAs were screened, and their potential biological functions were predicted by bioinformatics analysis. These results may bring new perspectives on the pathogenesis of D-T2DM and promote the development of new therapeutic approaches targeting RNAs.

## Data Availability Statement

All clean data have been submitted to Sequence Read Archive with accession number SRP274496.

## Ethics Statement

The studies involving human participants were reviewed and approved by the Ethics Committee of Beijing University of Chinese Medicine (BUCM) (2017BZHYLL0105). The patients/participants provided their written informed consent to participate in this study.

## Author Contributions

SG and GJ designed the experiments. BL and XB wrote the manuscript. PL and TA interpreted data and revised the manuscript. JL, YW, JZha, and XY performed experiments. TA, TW, JZhu, and YH performed data collection and analysis. All authors reviewed the manuscript and agreed to the publication of this article.

## Conflict of Interest

The authors declare that the research was conducted in the absence of any commercial or financial relationships that could be construed as a potential conflict of interest.
