# Supplementary material for: Transcriptome Sequencing Analysis of Peripheral Blood of Type 2 Diabetes Mellitus Patients With Thirst and Fatigue
Source: Front Endocrinol (Lausanne). 2020 Nov 9;11:558344. doi: 10.3389/fendo.2020.558344 (PMC7680858; doi:10.3389/fendo.2020.558344)
Supplement: Supplementary file 1 [file Table_1.DOCX]

**Supplementary table 1.** The results of quality control.

| **Sample name** | **Raw reads** | **Clean reads** | **Raw bases(G)** | **Clean bases(G)** | **Error rate(%)** | **Q20(%)** | **Q30(%)** | **GC**  **content(%)** | |
| --- | --- | --- | --- | --- | --- | --- | --- | --- | --- |
| LZC001 | 87845934 | 85181720 | 13.18 | 12.78 | 0.02 | 95.72 | 89.65 | 51.74 | |
| LZC002 | 87579572 | 84174478 | 13.14 | 12.63 | 0.02 | 95.79 | 89.72 | 54.3 | |
| LZC003 | 84227794 | 81076970 | 12.63 | 12.16 | 0.02 | 95.54 | 89.36 | 51.14 | |
| LZC004 | 81861938 | 79165804 | 12.28 | 11.87 | 0.02 | 95.64 | 89.55 | 51.37 | |
| LZC005 | 93403758 | 90275046 | 14.01 | 13.54 | 0.02 | 95.59 | 89.44 | 51.88 | |
| LZC006 | 85264928 | 81909328 | 12.79 | 12.29 | 0.02 | 95.29 | 88.92 | 51.84 |  |
| QYD1 | 94073262 | 91905938 | 14.11 | 13.79 | 0.03 | 97.67 | 93.45 | 54.01 |  |
| QYD2 | 108259870 | 104777872 | 16.24 | 15.72 | 0.03 | 97.86 | 93.9 | 54.37 |  |
| QYD3 | 92583772 | 90296430 | 13.89 | 13.54 | 0.02 | 98.08 | 94.35 | 57.78 |  |
| QYD4 | 100101444 | 97688440 | 15.02 | 14.65 | 0.03 | 97.58 | 93.33 | 58.02 |  |
| QYD5 | 99200116 | 97025894 | 14.88 | 14.55 | 0.03 | 97.97 | 94.16 | 57.11 |  |
| QYD6 | 102809758 | 94362122 | 15.42 | 14.15 | 0.03 | 96.13 | 91.21 | 73.18 |  |
