# Supplementary material for: Transcriptome Sequencing Analysis of Peripheral Blood of Type 2 Diabetes Mellitus Patients With Thirst and Fatigue
Source: Front Endocrinol (Lausanne). 2020 Nov 9;11:558344. doi: 10.3389/fendo.2020.558344 (PMC7680858; doi:10.3389/fendo.2020.558344)
Supplement: Supplementary file 2 [file Table_2.DOCX]

**Supplementary table 2(1).** The results of mapping analysis.

| **Sample name** | **LZC001** | **LZC002** | **LZC003** | **LZC004** | **LZC005** | **LZC006** |
| --- | --- | --- | --- | --- | --- | --- |
| **Total reads** | 85181720 | 84174478 | 81076970 | 79165804 | 90275046 | 81909328 |
| **Total mapped** | 81416961 (95.58%) | 80474891 (95.6%) | 76856758 (94.79%) | 75121159 (94.89%) | 85996771 (95.26%) | 77433979 (94.54%) |
| **Multiple mapped** | 15463611 (18.15%) | 20471532 (24.32%) | 12912655 (15.93%) | 12131077 (15.32%) | 16864804 (18.68%) | 13572868 (16.57%) |
| **Uniquely mapped** | 65953350 (77.43%) | 60003359 (71.28%) | 63944103 (78.87%) | 62990082 (79.57%) | 69131967 (76.58%) | 63861111 (77.97%) |
| **Reads map to '+'** | 32922526 (38.65%) | 30024251 (35.67%) | 31947846 (39.4%) | 31434299 (39.71%) | 34542095 (38.26%) | 31920898 (38.97%) |
| **Reads map to '-'** | 33030824 (38.78%) | 29979108 (35.62%) | 31996257 (39.46%) | 31555783 (39.86%) | 34589872 (38.32%) | 31940213 (38.99%) |
| **Non-splice reads** | 47053673 (55.24%) | 38403940 (45.62%) | 45467044 (56.08%) | 46751384 (59.06%) | 48093322 (53.27%) | 46075723 (56.25%) |
| **Reads map to exonic** | 56.28% | 70.37% | 60.68% | 52.72% | 60.27% | 59.18% |
| **Reads map to intronic** | 36.56% | 24.01% | 32.59% | 39.78% | 33.42% | 34.24% |
| **Reads map to intergenic** | 7.16% | 5.61% | 6.74% | 7.5% | 6.31% | 6.58% |

**Supplementary table 2(2).** The results of mapping analysis.

| **Sample name** | **QYD1** | **QYD2** | **QYD3** | **QYD4** | **QYD5** | **QYD6** |
| --- | --- | --- | --- | --- | --- | --- |
| **Total reads** | 91905938 | 104777872 | 90296430 | 97688440 | 97025894 | 94362122 |
| **Total mapped** | 88955649 (96.79%) | 101240149 (96.62%) | 88068800 (97.53%) | 94117018 (96.34%) | 94218887 (97.11%) | 82485434 (87.41%) |
| **Multiple mapped** | 17123718 (18.63%) | 18761462 (17.91%) | 26807023 (29.69%) | 24123776 (24.69%) | 19484850 (20.08%) | 36744881 (38.94%) |
| **Uniquely mapped** | 71831931 (78.16%) | 82478687 (78.72%) | 61261777 (67.85%) | 69993242 (71.65%) | 74734037 (77.02%) | 45740553 (48.47%) |
| **Reads map to '+'** | 35908921 (39.07%) | 41210052 (39.33%) | 30627113 (33.92%) | 34991012 (35.82%) | 37367180 (38.51%) | 22934010 (24.3%) |
| **Reads map to '-'** | 35923010 (39.09%) | 41268635 (39.39%) | 30634664 (33.93%) | 35002230 (35.83%) | 37366857 (38.51%) | 22806543 (24.17%) |
| **Non-splice reads** | 50670718 (55.13%) | 55975741 (53.42%) | 37304754 (41.31%) | 47761794 (48.89%) | 48368750 (49.85%) | 28925734 (30.65%) |
| **Reads map to exonic** | 58.32% | 59.04% | 76.72% | 52.49% | 68.12% | 32.03% |
| **Reads map to intronic** | 25.84% | 23.43% | 10.46% | 24.9% | 11.79% | 14.56% |
| **Reads map to intergenic** | 15.84% | 17.53% | 12.82% | 22.61% | 20.09% | 53.41% |
